# Social networks affect redistribution decisions and polarization

**DOI:** 10.1093/pnasnexus/pgaf339

**Published:** 2025-11-25

**Authors:** Milena Tsvetkova, Henrik Olsson, Mirta Galesic

**Affiliations:** Department of Methodology, London School of Economics and Political Science, London WC2A 2AE, United Kingdom; Santa Fe Institute, Santa Fe, NM 87501, USA; Complexity Science Hub, Vienna 1030, Austria; Santa Fe Institute, Santa Fe, NM 87501, USA; Complexity Science Hub, Vienna 1030, Austria

**Keywords:** inequality, redistribution, network effects, voting, polarization

## Abstract

We investigate theoretically and empirically how network structure affects collective decisions about redistribution in an unequal society. We study the effects of assortativity by wealth (observing others with similar or different wealth) and visibility by wealth (observing rich or poor others) on voting for redistribution and the polarization of votes, as well as satisfaction and perceptions of fairness. We develop a computational model and test the predictions of the model in an online network experiment. The results reveal that although most social networks lead people to under-observe inequality, different structural properties produce different collective outcomes: redistribution and polarization are the lowest in networks with maximum assortativity, where participants are segregated by wealth, and the highest in networks where the rich are most visible. Furthermore, segregation keeps the poor poorest but satisfied, while observing the rich makes them dissatisfied despite becoming better off. These findings suggest that political communication and policy strategies aiming to increase support for redistribution should enhance the visibility of excessive wealth. At the same time, it is crucial to ensure that this does not exacerbate polarization and conflict.

Significance Statement Most people dislike inequality, yet large disparities in income and wealth remain remarkably high in many democratic countries. One possible explanation is that people’s social networks affect their perception of inequality and consequently, their demands for redistribution. We present a computational model and an experiment with 1,440 participants to show that people under-observe inequality in homophilous social networks, with the rich more so than the poor, and under-observation leads to lower levels of redistribution, yet more contentment and harmony. When the poor over-observe the rich, they achieve more egalitarian outcomes but polarization and dissatisfaction increase. Our findings suggest that social networks pose an inherent and complex tradeoff between redistribution and political polarization.

## Introduction

In the last several decades, income and wealth inequality have increased in many countries, including established representative democracies with advanced industrial economies such as the United States, United Kingdom, Canada, and Australia (1–3). This trend presents a puzzle—if the majority of people dislike inequality (4, 5) and representative democracies respond to the majority vote, then redistribution policies should be implemented and inequality should decrease, not increase. One possible explanation for this puzzle is that democracy is failing due to the bundling of complex problems into rigid party ideologies, the corruption of the political elite, lobbying by the ultra-wealthy, public misinformation, the disenfranchisement of the poor, and other related issues (6–8). An additional explanation is that people habitually underestimate the amount of inequality in their society (9), as well as misunderstand their own position in the distribution (10–12). These perception biases are largely driven by the fact that people are surrounded by others with similar wealth (13, 14). If people do not observe inequality on a daily basis, they underestimate the severity of the problem and their own standing, and consequently do not take the due political stance and action. In this way, network structure can affect individual and collective decisions about redistribution, with consequences for inequality and political polarization.

In most human populations, wealth is distributed unequally and this has been so throughout human history (15, 16). Typically, the distribution of wealth is heavy-tailed, where the majority have little, while a small minority possesses a large proportion of all resources (17). Mathematically, this implies that the wealth of the median person is lower than the mean wealth in the population. The median voter theory posits that political decisions based on majority voting will reflect the preferences of the median voter (18–20). Thus, in an ideal democracy, the median voter, who has below-mean wealth, always selects redistribution and inequality decreases over time. This is a simplified baseline. In real societies, redistribution is also shaped by productivity concerns, institutional limits, and other systemic effects. Barring practical barriers to realizing an ideally democratic system, the logical veracity of this theoretical argument relies on two assumptions. First, individuals are expected to vote based on rational self-interest such that those with below-mean income always vote for redistribution because they are to gain from it. Second, individuals are assumed to possess accurate information about the income or wealth distribution of the entire population and their own position in it to be able to make an informed rational decision. Both of these assumptions have been challenged.

Self-interest, uncertainty, risk, and expectations undisputedly shape individual preferences for redistribution but so do cognitive biases, other-regarding preferences, and cultural factors. Compared to external observers, “involved decision makers” who get to benefit directly from their own redistribution decision prefer higher tax rates in experiments (21). Participants who are uncertain about their own position in the distribution (21, 22) or are at a risk of loss also prefer more redistribution (23), approaching it as a form of social insurance. At the same time, poor people who expect to rise up the income ladder tend to oppose high rates of redistribution (24). Yet, there is also evidence that loss aversion and status-quo bias drive disadvantaged individuals to oppose redistribution even when they benefit from it (25–27). Social preferences play a role too. People are generally averse to inequalities (28, 29) but will demand less redistribution when payoffs are earned by merit, via performance at a task, rather than determined by luck via a lottery (21, 30). Strong group identity could also trump own interests, whereby some participants would vote for redistribution when their own group is poorer than the outgroup even if they are rich, but prefer no redistribution when their own group is richer than the outgroup, even if they themselves are poor (31, 32). Racism also decreases support for redistribution when poverty is correlated with minority status and minorities are viewed as undeserving (33). Finally, cultural factors such as the belief in self-reliance, upward mobility, and negative effects from taxation on productivity appear to affect redistribution preferences too (34). Survey research has found that women, African Americans, the less educated, the more liberal, and those who come from countries with higher support for redistribution support more redistribution, while age has an inverted-U-shaped relationship with it (35). In short, preferences for redistribution are not only economically driven but also socially motivated and culturally tinted.

The assumption that people’s preferences for redistribution are based on accurate perceptions of inequality in their society has also been challenged. Measuring perceptions of economic inequality in society is difficult and scholars have explored different ways of asking survey responders about it. Although some researchers find that responders’ estimates approximate the actual income distribution on average (36), most confirm that people tend to underestimate the overall level of inequality (9, 37, 38). Furthermore, people misperceive where exactly they stand in the distribution: for instance, studies with Swedish and Dutch samples suggest that these participants underestimate how rich they are, with the better off particularly prone to self-depreciation (12, 39, 40). American, Spanish, and Danish samples have been found to veer towards the middle (10, 11, 41), with self-depreciation appearing again for the better off but self-enhancement for the worse off responders. The most prominent explanation for these biases is that people use a self-centered perspective and form their opinions based on nonrandom reference groups (42). Specifically, people’s social circles are not representative of the general population but tend to be similar to them in income and wealth. People extrapolate population estimates from these homogeneous samples and even though they are not naive and partially correct for the bias (40), they still end up underestimating inequality, with the economically advantaged more prone to underestimation than the worse off (14). Given the right-skewed distribution of income and wealth, more successful individuals would be overrepresented among people’s contacts, making the average individual feel worse off than they actually are (40). The better off may be particularly prone to self-depreciation, perceiving their position to be worse than it actually is. This dependence on local social information for gauging societal inequality suggests that the very structure of social networks could have profound implications for the distribution of resources. Indeed, theoretical work has shown that network structure can constrain the levels of inequality that are sustainable, emphasizing the potential for social connections to shape economic outcomes (43).

To summarize, people’s preferences for redistribution are dictated by social considerations, in addition to self-interest, and based on perceived, rather than actual, inequality of the population. This implies that people’s perception of inequality can be manipulated to change their behavior. However, such manipulations have produced inconsistent results. Field experiments with poor-looking confederates in affluent neighborhoods and luxury cars parked in poor neighborhoods show that exposure to inequality decreases affluent individuals’ willingness to redistribute (44) but raises the poor’s support to tax the wealthy (45). Similarly, correcting low-income responders’ misperceptions by informing them that they are poorer than they believe increases their support for progressive taxation (10, 11). Yet, others find that information about actual income standing does not change preferences for redistribution (46).

In this article, we manipulate individual wealth and network structure to alter individuals’ preferences for redistribution and investigate when even a direct democracy that follows the median-voter rule with referenda may fail to reduce economic inequality. We focus on how network assortativity and visibility by wealth affect voting for redistribution via taxation. These network properties model the effect of friendships, organizational structures, news, and social media on people’s perception of society and their own position in it. More generally, we study how social structure influences individual perceptions and preferences and how these preferences, when aggregated through majority voting, determine collective outcomes. This macro–micro–macro approach parallels two previous studies. Similarly to Ref. (47), we investigate the effects of network assortativity on voting outcomes, but we focus on inequality and redistribution instead of party coordination. In parallel to Ref. (48), we study the effects of network assortativity on inequality but we assume collective decision making rather than unilateral transfers.

We develop a computational model simulating a population with unequal wealth distribution, where individuals who are inequity averse with respect to their neighbors vote on tax rates for redistribution. By varying network assortativity and visibility by wealth, we explore how these factors influence perceived inequality and voting outcomes. We then conduct an online experiment to test the model’s predictions across several network structures. We investigate how these structures affect the selected tax rates, the polarization of votes, satisfaction with one’s own score, and perceptions of the fairness of scores in the group.

## Model

The model assumes a large population of size N=200 with unequally distributed wealth, drawn from a right-skewed beta distribution. Individuals observe a subset of the population of size n=8 (their social network) and vote for a redistribution tax rate that maximizes their utility with respect to their wealth compared to the wealth of those they observe. Individuals are inequity averse and while they appreciate higher personal wealth, they dislike both disadvantageous and advantageous inequality. This is modeled with a utility function that increases with one’s wealth but decreases when one has less than others and, to a lesser extent, when one has more than others (49). Votes are cast in a referendum (29) and the median vote wins. Individuals pay the selected percent of their wealth as tax and the collected tax revenue is then redistributed equally among all. Thus, the richer contribute tax of higher absolute value than the poorer but everyone receives the same benefit. The model’s setup and parameter choices are detailed in the Materials and methods section.

We use a preferential attachment process (50) to create links and manipulate network structure. The process is defined by parameters of assortativity and visibility by wealth and determines the composition of the observed subset. Assortativity refers to the tendency of individuals to observe others with similar or dissimilar wealth. Positive assortativity will produce homophilous networks, while negative assortativity, or dissortativity, will lead to heterohphilous networks. Visibility determines whether the rich or the poor are more likely to be observed. We assume a directed network, meaning that observation is asymmetrical. Our model extends existing network models of perception bias (14, 51–53) by combining directedness with simple and intuitive parametrization for homophily/heterophily by continuous attributes, as well as adding a new parametrization for attribute-based visibility.

Our comprehensive investigation shows nontrivial interactions between wealth assortativity (and the resulting homophily) and visibility by wealth (Figs. S1 and S2). Overall, inequality is underestimated, in particular by individuals with more rich social contacts, and by poor individuals in heterophilous networks (Fig. S3A). The visibility of the rich is more impactful than the visibility of the poor. Because the rich are few in the population, over-sampling them is more consequential than over-sampling the poor, who are already more visible simply because they are numerous. More importantly, while a single rich person dramatically increases the observed differences in wealth in one’s social circle, several rich people decrease them. Over-observing the rich gives the wrong impression that wealth is more evenly distributed in the population than it actually is.

Besides perceived inequality, wealth assortativity and visibility affect also preferences about the tax rate. The preferred tax rate depends on the expected utility of redistribution (see section on Individual decision-making in Materials and methods). The higher the number of rich people in one’s social circle, the larger the perceived tax base and consequently, the higher the potential personal gain of taxes. The higher the number of poor people, the smaller the perceived tax base and the smaller the tax proceeds. Thus, visibility of the rich induces shared agreement on higher redistribution, resulting in both higher selected tax rate and lower polarization (measured as the mean absolute deviation of votes). In contrast, visibility of the poor results in lower selected tax rate but higher polarization, especially under heterophily, when the poor get to observe more rich others (Fig. S3B).

**Fig. 1.**
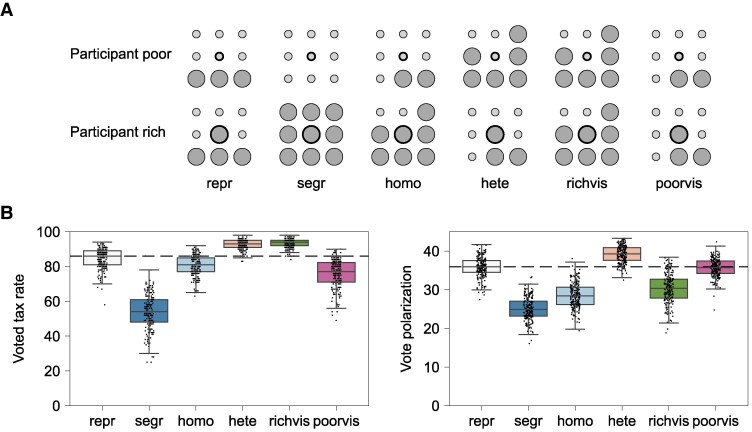
Treatments and model predictions for the experiment. A) The reference samples observed by the rich and the poor in the six experimental treatments: representative (repr), segregated (segr), homophilous (homo), heterophilous (hete), rich visible (richvis), and poor visible (poorvis). Each group in the experiment consists of 24 participants, 15 of whom are randomly assigned to be poor and nine—rich. Each participant (the circle in the middle with thick outline) observes the score of eight others and who these others are depends on the participant’s status (small light gray circles for poor, large dark gray circles for rich) and the six treatments. B) Model predictions for the expected differences in the voted tax rate (the median vote) and vote polarization (the mean absolute deviation of votes) between the six experimental conditions we test. Black markers show data points from 200 simulation runs. The horizontal dashed lines show the expected values in the control condition (repr) where each individual observes a representative sample of the group.

To generate testable hypotheses for an experimental setup with human participants (see Experiment section), we next implement the model on a smaller population of 24 individuals, nine of whom are rich and 15 poor. We investigate six fixed network structures: representative, segregated, homophilous, heterophilous, rich visible, and poor visible (Fig. 1A). Each individual observes the wealth of eight others. In the representative network, everyone observes three rich and five poor, which corresponds to the ratio of rich to poor in the group. In the homophilous network, the rich observe six rich and two poor and the poor observe two rich and six poor, representing a tendency to over-observe others of similar wealth; in the heteorophilous network the ratios are reversed such that the rich over-observe the poor and the poor over-observe the rich. In the rich-visible network everyone observes six rich and two poor, while in the poor-visible network everyone observes two rich and six poor. Finally, in the segregated network, the eight others the rich observe are all rich and the eight others the poor observe are all poor.

We use the qualitative differences between the model outcomes for the six network structures to derive predictions for the experiment. Compared to the control condition with representative samples, we expect higher voted tax rate in the networks with heterophily and visibility of the rich but lower voted tax rate in the networks with homophily, visibility for the poor, and segregation (Fig. 1B). The lowest tax rates are expected to occur in the segregated networks, because the poor might think the tax base is small and the rich might think that there are few beneficiaries of redistribution. Because the rich and poor agree on low taxes, albeit for different reasons, we also expect lower vote polarization in the networks with segregation and homophily compared to the control condition with representative samples. Similarly, polarization is lower when the rich are visible but in this case the mutual agreement is for higher taxes. In the rich-visible condition, poor agents observe richer neighbors and consistently vote near 100%, leading to very low variance within their group. Rich agents, on the other hand, may observe some neighbors who are slightly poorer than themselves and hence, a small tax rate will still yield a net benefit. This results in more dispersed votes among the rich but smaller inter-group distance between the rich and the poor compared to the heterophilous condition. Thus, the overall polarization score is lower in the networks with rich visible in contrast to the networks characterized by heterophily, where the rich vote consistently low as they observe more beneficiaries than contributors. In other words, we expect that when the rich are visible, the group achieves both low inequality and high agreement on the preferred level of redistribution. We expect that homophily and segregation similarly reduce vote polarization but fail to reduce inequality. The pre-registration protocol for the study included these hypotheses about the effects of network structure on the voted tax rate and vote polarization, in addition to exploratory analyses about the effects of network structure and group outcomes on individual satisfaction and perceptions of fairness.

## Experiment

Participants interacted in groups of 24, with nine participants randomly assigned an initial score of ∼200 (the “rich”), and the remaining 15 participants assigned an initial score of ∼20 (the “poor”). The actual initial score varied in fixed steps within +/−10% for each subgroup (rich or poor) in order to make the binary distinction less obvious from the participants’ perspective. The instructions informed participants that they are part of a group of 24 but can only observe the score of eight others. Participants were also explicitly informed about the range of initial scores in the entire group and the median-voter rule (Fig. S4). We used a “context rich” setting by employing language and framing around redistribution/tax. This approach is often used in experiments on tax compliance (54, 55) and framings for taxation (56). Participants interacted asynchronously over three rounds. In the first round, they used a slider to select a tax rate and press a button to cast their vote. After 24 hours, they were invited again to view the vote results from the previous round and adjust their vote. The decision screen after the first round depicted the collectively selected tax rate, the participant’s and their neighbors’ initial scores, and their post-tax scores (Fig. S5). After the three decision rounds, participants were invited one last time to view the final result and complete a brief survey with questions about their demographic characteristics, satisfaction with their score, perception of the fairness of the scores in the group, rationale for voting, and perception of the group decision.

**Fig. 2.**
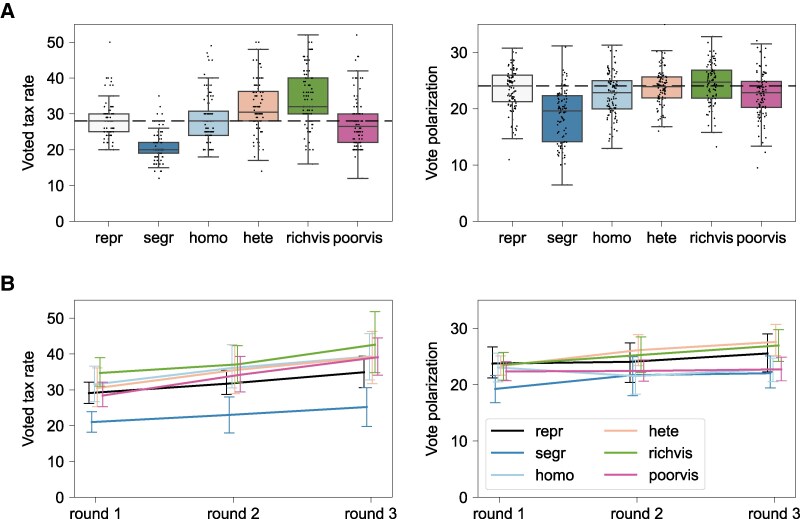
Experimental results for the voted tax rate (the median vote) and vote polarization (the mean absolute deviation of votes). A) Results in the first round. Black markers show data points from 100 statisticized groups for the representative (repr), segregated (segr), homophilous (homo), heterophilous (hete), rich visible (richvis), and poor visible (poorvis) treatments. The horizontal dashed lines show the median value in the control condition where each participant observes a representative sample of the group (repr). B) Result changes over the three rounds for the ten groups in each treatment that interacted repeatedly. Plots show means and 95% CI.

We recruited 1,440 participants from Prolific and assigned them to one of 60 groups, with ten groups per network structure. In the first round, the individual decisions are independent and hence, we repeatedly sample participants based on poor/rich status and observation condition into fictional groups to estimate group-level outcomes. We visualize the first-round results from 100 such “statisticized” groups per treatment (Fig. 2A) but in Tables S1 and S2, we report statistical tests estimated for the ten groups per treatment that interacted over three rounds (Fig. 2B).

The experimental results in Fig. 2 qualitatively align with the model predictions in Fig. 1 with some notable exceptions. As predicted, the segregated network has significantly lower voted tax rate than the other treatments (Fig. 2A), and this effect persists throughout the three voting rounds (Fig. 2B, Table S1). Also as predicted, the poor-visible treatment starts with a lower voted tax rate than the rich-visible treatment in the first round, although the two conditions become more similar later on. Similarly, as predicted, the segregated network condition starts with a significantly lower vote polarization than the heterogeneous and rich-visible networks and these differences persist until the end (Table S2). Polarization decreases in the homophilous and poor-visible networks, while it increases in the heterogeneous and rich-visible networks, with the differences becoming significant in the third round. The higher vote polarization in the heterophilous network compared to the segregated and homophilous networks matches the predictions, but the difference in vote polarization between the poor-visible and rich-visible networks is in the opposite direction.

**Fig. 3.**
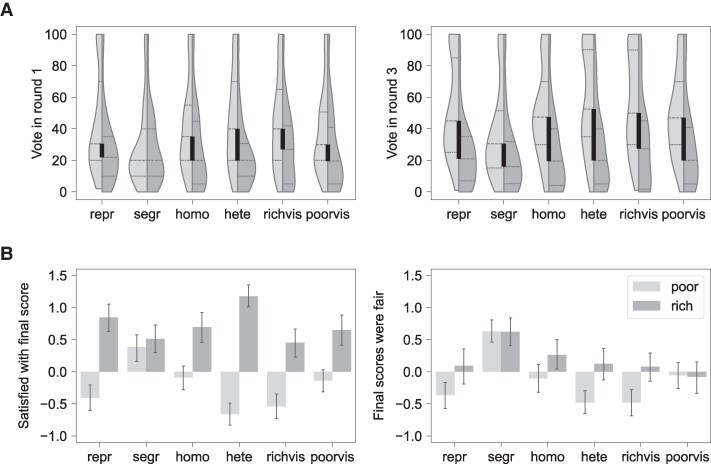
Polarization between the poor (light gray) and the rich (dark gray) in the six experimental treatments: representative (repr), segregated (segr), homophilous (homo), heterophilous (hete), rich visible (richvis), and poor visible (poorvis). A) Voting patterns in round 1 versus round 3. Plots show kernel density estimates of the distribution of votes, with the black vertical bands depicting the difference between the median for the rich and poor. B) Satisfaction and fairness perception at the end of the experiment. Plots show means and 95% CI.

In addition to general disagreement among voters, we examine polarization as the divergence between the poor and the rich (57) with regard to voting patterns, self-reported satisfaction with own score, and self-reported perception of the fairness of the scores in the group (Fig. 3). Over subsequent rounds, the vote differences between the rich and poor increase but only because the poor increase their vote, likely as a result of observing the benefits of higher taxation; in contrast, the rich stay their course (Table S3). Most notably, the poor radicalize when they observe a majority of rich others in the heterogeneous and rich-visible networks: many of them vote for 100% taxation (Fig. 3A). The poor also feel the least satisfied with their own score and the fairness of the final score distribution in these networks (Fig. 3B), despite being relatively well off in the end. In stark contrast, segregation keeps the poor poorest yet satisfied, while observing a majority of similarly poor others in the homophilous and poor-visible networks makes the poor indifferent and apathetic. These observations are confirmed by the open-ended answers in the exit survey: participants assigned to be poor are most likely to mention conformity to the majority vote as their strategy in the homophilous, poor-visible, and segregated networks (Fig. S6) and most likely to judge the final outcomes as unfair in the heterophilous and rich-visible networks (Fig. S7).

The data confirm the assumption of rational self-interest in our model: in the first round, the poor vote for higher taxes than the rich except in the segregated networks, where they vote similarly (Fig. 3A, Table S3). The free-text survey responses echo the prominence of self-interested strategies, but also suggest that participants were equally concerned with equality, fairness, and balance, confirming the inequity aversion assumption (Fig. S6). Nevertheless, the data point to several other potentially relevant behavioral mechanisms that our model does not account for.

First, participant behavior in the experiment appears to be dictated by strong priors about taxation. To begin with, most voting decisions gravitate to the actual level of taxation in the United States (effective rate at 20–25% for most residents), resulting in much lower taxation rates than the rationally optimal ones predicted in the model. Conservatives vote for lower taxation, while those who perceive themselves in a lower income percentile and prefer a higher taxation level in the US vote for higher tax in the experiment; interestingly, it is the perception of socioeconomic rank that matters, not the actual household income or education (Table S4). Further, women tend to vote for lower taxation, which seemingly contradicts prior survey results indicating that they prefer higher levels of redistribution (35, 37, 58). Upon inspection, we find that this effect is entirely driven by women assigned to the “poor” condition in the experiment (Table S4, Fig. S8). There are several possible explanations for this effect. Women had more difficulty understanding the experiment instructions (1.9 attempts to answer the instructions quiz correctly versus 1.7 for men, with Wilcoxon rank sum test p=0.001) and thus possibly misunderstood the benefits of redistribution for the poor in the specific experiment. Additionally, women reported that they were less likely to act out of self-interest but more likely to conform to others’ decisions and exogenous norms (Fig. S9), potentially making them less willing to object to their arbitrarily assigned disadvantaged status in the experiment and more likely to follow the widespread norm in US society that high taxes are undesirable.

Second, there is also evidence for strategic voting, whereby participants adjust their votes in response to the median vote. Some participants vote by conviction, not changing their choice over rounds, but others appear to conform to the median vote, choosing not to diverge too much from the group; still others appear to double-down and shift their vote further away from the median, likely with the hope to influence the group outcome (Fig. S10). In sum, voting in the experiment was not simply a matter of constrained preferences but was also normative, tied to exogenous beliefs and experiences, as well as strategic, aimed at reducing group dissonance or influencing group decisions.

## Discussion

Information via close social contacts, anonymous daily encounters, public media reports, and social media content affects people’s perception of their relative position in the socioeconomic status hierarchy and of the severity of socioeconomic disparities in society. In turn, these perceptions affect people’s redistribution preferences. If people are presented with biased information, they may fail to realize better outcomes for themselves and others. And if people have information and preferences that clash with the collectively agreed-on level of redistribution, they may fail to appreciate political compromises and personal gains. We presented a model and an experiment that investigate these scenarios.

In the model, redistribution increases when the poor observe many rich others (in the heterophilous and rich-visible networks) but decreases when they observe many others who are just as poor (in the segregated, homophilous, and poor-visible networks). The lower levels of redistribution under homophily and segregation occur because both the poor and the rich underestimate inequality and the benefits of redistribution; thus, homophily increases consensus. In contrast, the higher levels of redistribution under heterophily come at the cost of higher polarization: the poor demand more from the rich they see, while the rich become more protective of their advantage. Interestingly, when the rich are more visible to all, polarization decreases because the rich are unaware of the poor majority and less protective of their wealth. The model suggests that increasing the visibility of extreme wealth, for example through news reports, political discourse, or social media, can raise support for redistribution. This prediction focuses on the role of social information and does not account for trade-offs between redistribution and economic efficiency. In real societies, concerns about reduced productivity, lower innovation, or increased tax avoidance may limit support for high taxation, even when inequality is clearly visible.

Unlike in the model, however, in the experiment, polarization in the rich-visible network did not decrease but increased. This occurred because the rich did not increase their support for redistribution at all, while the poor radicalized to demand 100% taxation. Thus, the empirical results suggest that the poor observing the rich increases polarization, while the poor observing other poor could lead to acceptance and apathy. The latter decreases polarization but does not improve redistribution and in the case of absolute segregation—worsens it. Social networks and the unequal distribution of wealth appear to generate a tradeoff between the level of redistribution and agreement about it, with no simple resolution.

The experiment was intentionally framed in terms of taxation to simplify the instructions for participants and increase external validity. However, this came at the cost of internal validity. Most notably, participants came with strong priors about taxation, making them less sensitive to the experimental manipulations. Nevertheless, over the subsequent rounds, as they obtained evidence of the benefits from redistribution, the poor started to demand higher redistribution universally across all networks. Notably, the rich did not attempt to compensate for the increasing taxation rate by veering towards the lower end: many recognized the tradeoffs and the need to balance different motivations and goals to achieve fairness (Fig. S6). Thus, it appears that pre-existing conceptions about redistribution are malleable and more importantly, malleable in an asymmetrical way such that evidence of benefits boosts support among the beneficiaries but incurring losses does not affect those who know they have the most to lose. This also means, however, we should not expect support for higher redistribution to come from the rich—empirically, this did not occur in any of the networks, in contradiction to the model predictions.

Our study falls within a large and growing literature investigating the effect of social networks on socioeconomic inequality in society (59, 60) with both observational data (61, 62) and controlled experiments (48, 63–67). We integrate three different aspects of the problem of redistribution that previous studies from network science, psychology, and economics have approached independently. First, we build on computational models that investigate the effects of network structure on perception bias (14, 51–53) to study how social structure influences individual perceptions of economic inequality. Second, starting from cognitive and social psychological research on inequality, we shift focus from individual perceptions and preferences (9–11) to individual decisions and their collective outcomes. We do this by employing utility models of individual preferences for redistribution and fairness (29, 49) to translate social information to decisions. Third, we borrow from experiments of voting for taxation (20, 68, 69) to aggregate individual votes to collective decisions and outcomes under different network structures.

Note that we use the model to derive only effect-direction predictions for the differences between the different network structures; generating exact point and effect size predictions would require a more sophisticated cognitive model that has been carefully calibrated with behavioral data. For instance, in this study, we used crude prior estimates of the variation in the aversion to advantageous and disadvantageous inequity in a generic population. Instead, future research could use the strategy method to estimate these parameters for the individuals in the study sample and then use the estimates in the simulations to obtain quantitative predictions at the group level. This would offer more direct evidence as to whether women object less to disadvantageous inequity. While survey research of the general US population suggests that women prefer higher levels of redistribution (35, 58), here we incidentally discovered that women who are randomly assigned to have less than others systematically vote for lower taxation than men in the same position; the only time women vote for more taxation than men is when they are rich in homophilous networks. Future research should explore whether the seeming discrepancy is because women’s voting decisions differ from their stated preferences or because women are less likely to perceive relative deprivation and move in more homophilous social circles.

The strategy method could also clarify whether the gap between our model and experiment is due to parameter assumptions or missing mechanisms. If the latter, the cognitive model could be extended to include risk aversion, loss aversion, aversion to group inequality, group identity correlated with poverty (70), biases in inference from social sampling, and strategic voting. Another promising model extension is to also account for individual satisfaction and perception of fairness. Finally, an interesting extension would be to make the network structure endogenous, for example, by using a model that allows for bilateral link formation and unilateral link cuts.

Further, we acknowledge that some of the model-experiment discrepancies could be due to limitations of the experimental design or peculiarities of the participant population. Future research should investigate whether the results replicate if the decision situation is framed in neutral terms, without references to taxation, and if the experiments are conducted with populations sampled from countries with more public support for redistribution than the United States. Prior research suggests that the selected taxation rate will be higher (35, 56), but whether it differs between social networks as we observed here remains an open question.

Necessarily, our study relied on a very simplified version of a political economy, with a flat taxation rate, referendum voting, and equally shared benefits. This departs from the reality of many modern societies, which have progressive taxation, complex political representation systems, and complex benefit generation and distribution arrangements with investment and innovation growth, administrative inefficiencies, discrimination, biases, and fraud. Our results show that even under simplified and ideal conditions, redistribution and agreement about it are susceptible to social information and interaction networks. In particular, biased information from social networks and media could affect social perception and stifle political action. Our findings suggest practical applications for political communication and policy. For instance, we can increase support for redistribution by increasing the visibility of extreme wealth via news reports, political discourse, and social media discussions. However, we need to make sure this does not exacerbate polarization and conflict. At the same time, it is important to acknowledge that polarization and conflict are not necessarily negative. In fact, the absence of conflict in a highly unequal economy may indicate deeper issues, such as segregation or the dominance of pro-wealth ideologies. In such cases, it is complacency, rather than conflict, that should be avoided.

## Materials and methods

### Model

The model assumes that a population of *N* individuals observe the wealth $x_{i},\ldots ,x_{n}$ of a fixed sample of size *n* of the population and vote for the redistribution policy (tax rate *τ*) that maximizes their utility. What we refer to here as wealth could represent the value of income, financial assets, or property, possibly adjusted for regional variation in the cost of living and local taxes, net of the personal exemption and standard deduction, etc.

#### Network structure

To generate a network, we iterate over all individuals and create links to *n* others, where with some probability *p* these links are created preferentially with regards to others’ wealth or wealth similarity and with probability 1−p the links are randomly allocated. We model assortativity by wealth by letting with p=|h| links be drawn with relative weights of $w_{ij}=|x_{i}-x_{j}|$ for heterophily and $w_{ij}=\max\Delta x-|x_{i}-x_{j}|$ for homophily, from all possible others j≠i. The parameter −1≤h≤1 determines the strength of homophily (h>0) or heterophily (h<0). We model visibility by wealth by letting with p=|v| links be drawn with relative weights of $w_{j}=x_{j}-\min x$ for prominent wealth (v>0) and $w_{j}=\max x-x_{j}$ for prominent poverty (v<0), where the parameter −1≤v≤1 determines the strength of the effect. When both assortativity and visibility play a role, we multiply the weight for wealth proximity/distance by the weight for the other’s wealth/poverty in the preferential attachment component.

The model builds on and extends several existing network models of homophily for studying perception bias. Similarly to Ref. (51), we model a directed network but we allow for homophily for continuous attributes (wealth, income, attractiveness, etc.) rather than binary or categorical characteristics (gender, race, educational qualifications, etc.). Reference (14) also models homophily by wealth but uses exponential weighting for the link probabilities rooted in economic discrete choice models (71), while we use simpler, more intuitive weighting related to preferential attachment models from physics (50). Our model, however, does not assume preferential attachment by degree as (51–53) do but rather, manipulates preferential attachment by attribute with the visibility parameter; long-tailed degree distributions are not given but can emerge under some conditions (Fig. S2).

Because the model is simplified to zoom into the effects of interest, it produces several network properties that are not realistic. For instance, the model assumes a fixed outdegree, but prior research has shown that the rich have larger (72) and more diverse (73) social networks. The model also assumes population-level homophily and visibility parameters that do not depend on individual wealth; yet, there is empirical evidence for higher homophily among the wealthiest, as well as an upward bias for the middle classes (74). Finally, in the model, assortativity depends on absolute income differences, but a logarithmic function may be more appropriate since small differences matter less at higher values (14). We erred on the side of simplicity here but model extensions are certainly possible to account for these additional effects. The model can also be adapted to use empirical social networks or highly stylized fixed networks as the ones we employ in the experiment.

#### Individual decision-making

Following (49), we assume that the utility of person *i* is $U_{i}(x)=x_{i}-\alpha_{i}\frac{1}{n}\sum_{\;j=1}^{n}\max\{x_{j}-x_{i},0\}-\beta_{i}\frac{1}{n}\sum_{\;j=1}^{n}\max\{x_{i}-x_{j},0\}$, where $\alpha_{i}$ and $\beta_{i}$ are individual parameters. Utility depends on absolute wealth but is decreased by both disadvantageous and advantageous inequality with respect to the reference group *n* and more so by the former ($\alpha_{i}\geq \beta_{i}$).

As per Ref. (29), a redistribution policy *τ* subtracts tax $t_{i}=\tau x_{i}$ and adds benefit $b=\frac{1}{N}\sum_{k=1}^{N}t_{k}$ to *i*’s wealth, for all *i*’s. However, we assume that when making a voting decision, individuals only account for the neighbors they observe and hence, the potential benefit is calculated over *n*, rather than *N*. For simplicity, the utility model we employ assumes that individuals do not correct for social circle perception (39, 40). That is, individuals are not aware that their sample is biased and use it directly as a basis for the population estimate. Further, as (29) note, this is a model of “self-centered inequality aversion” and it does not include objective inequality, that is, it does not consider the individual’s ideal fair distribution regardless of their own position in it. Model extensions that account for sampling bias compensation and aversion to objective inequality are possible in future research. Similarly, the decision-making model can be easily extended to account for risk and loss aversion.

Before voting for a tax rate, individuals estimate their expected utility for all possible whole-percent tax rates [0,100] based on their own wealth and the wealth of the *n* other agents they observe. They then select their vote with probability according to the softmax function $p_{c}=\frac{e^{\gamma U_{c}}}{\sum_{\tau =0}^{100}e^{\gamma U_{\tau }}}$. This function, commonly used in reinforcement learning (75, 76), implements probabilistic choice by heavily weighing the maxima as determined by the parameter *γ*.

To determine the agreed tax rate, the median vote is selected and then implemented. In the case of an even population size, the median is estimated as the whole-number rounded average of the two middle votes. This voting procedure, successfully employed in previous studies (20, 30, 77), is simple to implement and intuitive to explain to experiment participants.

#### Model parameters and analysis

For the utility function, we draw individual-specific $\alpha_{i}$ and $\beta_{i}$ according to empirical estimates of their value and prevalence in the population: for *α*, 30% of the population are estimated to have a value of 0, 30%–0.5, 30%–1, and 10%–4; for *β*, 30% are estimated to have a value of 0, 30%–0.25, and 40%–0.6 (49). Since the estimates for the two parameters were obtained from different individuals in different roles, the correlation between $\alpha_{i}$ and $\beta_{i}$ is unknown. Therefore, for now, we assume the two values are independent. Additionally, we assume that the individual’s values are fixed and exogenous to the wealth distribution the individual observes and the individual’s own position in that distribution. For the probabilistic vote choice, we set the parameter γ=0.1. The value was chosen to introduce enough variation in individual decisions in order to detect differences between the different network structures; with γ=0 structure plays no role because voting decisions are completely at random while with γ>0.5 the outcomes are binary because the decisions are highly sensitive to small variations in the network structure.

We conduct simulations with two versions of the model. In the first version, we model a large population and a realistic wealth distribution: population size N=200, sample size n=8 for each agent, and a right-skewed wealth distribution drawn from the beta distribution with alpha=1, beta=6 and values divided by the distribution mean of alpha/(alpha+beta) and multiplied by 100 to obtain an expected mean wealth of 100 in the population. We confirmed that the model results are consistent for larger population and sample sizes.

The second version modifies the model to closely match the setup of the experiment. We set the population size N=24 and the sample size n=8, and assign 9 agents to be rich with wealth of about 200, and 15 to be poor with wealth of about 20, where the exact wealth varies in fixed steps within +/−10% for each group (rich or poor). This variation is introduced in the experiment to prevent participants from second-guessing the purpose of the study and to weaken the experimenter demand effect. The Gini coefficient for the initial wealth inequality is thus set to 0.519. We fix six different structures for the observation network to test: representative, segregated, homophilous, heterophilous, rich visible, and poor visible (Fig. 1A).

We present simulation results based on 100 repetitions (200 for the model matching the experimental setup). We analyze how network structure affects the level of inequality that the poor and the rich observe, the voted tax rate, and the level of polarization of vote decisions. We measure inequality with a Gini estimate that adjusts for sample size: $G=G^{L}\frac{n}{n-1}$, because the original Lorenz-based coefficient $G^{L}$ is downward biased for small populations. This adjustment reformulates the Gini coefficient with an emphasis on the differences between individuals, or “experienced inequality” (78), which corresponds to our focus on individual perception and network structure. We measure vote polarization with the mean absolute deviation of votes, which tells us how spread out the votes are (57, 79). In the Supplementary information, we replicate some of the analyses with two alternative dispersion measures: the variance and the kurtosis.

### Experiment

The experiment was implemented in the Python web development framework Django. Participants were recruited from Prolific, where the recruitment pool was restricted to US residents. When they logged into the experiment website, participants read a brief tutorial, followed by three multiple-choice questions about the game rules to test their attention and understanding. Participants could not proceed to the game until they answered all questions correctly, with an unlimited number of attempts to do so. After completing the three voting rounds, participants were invited one last time to view the final results and complete a survey about their basic demographics (age, gender, race and ethnicity, household income, perceived income percentile, ideal tax rate, education, religion, and political orientation), the rationale for their voting decisions, their satisfaction with their initial and final score, their perception of the fairness of the initial and final scores in the group, and their perception of the group decision. The questions about decision rationale and group perception elicited free-text answers, which we manually labeled into categories (see SI Text, Tables S6 and S7).

Using power analysis, we estimated that we required 10 observations per treatment to detect the expected small effect of 20% difference in means between two treatment conditions at the 0.05 significance level with a power of 85%. The experiment design was approved by the Research Ethics Review Board of the London School of Economics and Political Science (LSE Ref.329366). Informed consent was obtained from all participants. After conducting a pilot study, we collected data in three batches over the first 3 weeks of May 2024. Participants were paid £1.50 for completing the first round and, as long as they participated at least once more, a bonus based on their final score at the rate of 100points=£2.50. For the 89% who returned at least once after the first round (see Fig. S11 and Table S5), the bonus varied between £0.62 and £5.17 with a mean of £2.17. Participation was spread over 4 days but activity took <10 min combined, resulting in a mean pay well above the minimum wage in any US state.

For the statistical analyses, we use the Mann–Whitney *U* test to compare the collective outcomes between treatments. This is a nonparametric test of the null hypothesis that a randomly selected value from one treatment is equally likely to be less than and greater than a randomly selected value from another treatment. With six treatments, we conduct 15 pairwise comparisons. Since the tests are hypothesis-driven and the 15 comparisons are nonindependent, we do not correct for multiple testing. In the first round, the individual decisions are not group-dependent and hence, we can randomly assign individuals to groups according to poor/rich status and the observation condition they experience (Fig. 1A); this allows us to generate a large number of synthetic groups and compare the trends in the experiment to those from the model. Additionally, we conduct individual-level analyses to test the behavioral assumptions in the model and explain the observed outcomes. For these exploratory analyses, we use mixed-effects regression models with fixed effects for batch, group, individual, and round, whenever relevant.

## Supplementary Material

pgaf339_Supplementary_Data

## Data Availability

The preregistered protocol is available at https://doi.org/10.17605/OSF.IO/8VYBS. Anonymized experimental data, model code, and analysis scripts are available on Figshare at https://doi.org/10.6084/m9.figshare.28676018.v3 (80).

## Preprints

A preprint of this article is published at https://doi.org/10.31219/osf.io/bw7ux.
