# Supplementary material for: Social networks affect redistribution decisions and polarization
Source: PNAS Nexus. 2025 Nov 25;4(11):pgaf339. doi: 10.1093/pnasnexus/pgaf339 (PMC12645457; doi:10.1093/pnasnexus/pgaf339)
Supplement: pgaf339_Supplementary_Data [file pgaf339_supplementary_data.pdf]

Supplementary materials for:

Social networks affect redistribution decisions and polarization

Milena Tsvetkova, Henrik Olsson, and Mirta Galesic

Model results

We analyze the model (for a description, see Materials and Methods in the main text) to obtain a qualitative understanding of the effects of unequally distributed wealth and network structure on perceptions of inequality and voting for redistribution. The model results are based on a population size of 200 and 100 repetitions for each parameter combination, which are sufficient to represent long-tailed distributions of wealth, model complex network structures, capture the variation for a specific parameter combination, and reveal visible differences between parameter combinations. We confirmed that the model results are consistent for larger population (N=1000) and sample (n=20) sizes. The results from these additional simulations are available in the analysis files on Figshare at https://doi.org/10.6084/m9.figshare.28676018.v3 [1].

Due to the skewness of the wealth distribution (many poor, few rich), the parameters for homophily (h) and visibility (v) and their interaction result in non-trivial patterns of wealth assortativity (as reflected in the similarity of own and neighbors' wealth, Fig.S1) and visibility by wealth (as reflected in individuals' indegree Fig.S2). Similarly, because agents at different positions of the wealth distribution experience different sampling restrictions within their network, their local perceptions of inequality deviate from global inequality in complex ways (Fig.S3A). Overall, homophily and visibility for the rich independently cause inequality to be under-observed. Consistent with [2], under homophily, the rich under-perceive inequality more than the poor. Under heterophily, it is the poor who under-perceive inequality more than the rich. Notably, increased visibility for the rich reduces perceived inequality for all, whereas increased visibility for the poor has no effect. Increased visibility of the poor has no effect because the poor are already over-represented in the population. In contrast, oversampling the handful of rich individuals noticeably increases the homogeneity, and hence decreases the inequality, of the observed samples. Over-observation

occurs only under restricted conditions and with limited impact: the lower middle class will over-perceive inequality but only when both heterophily and higher visibility for the poor are present.

The individual perceptions of inequality result in different individual votes and collective decisions (Fig.S3B). Homophily and higher visibility of the poor decrease the selected level of redistribution, while heterophily and higher visibility of the rich increase it. Compared to the random network without structure, higher visibility of the rich always leads to higher voted tax rate. Higher visibility of the rich also decreases polarization – it results in the lowest level of disagreement around the selected taxation rate. In contrast, higher visibility for the poor lowers redistribution but increases polarization. Interestingly, heterophily can counteract the suppressing effect of visible poverty on redistribution but at the cost of even higher polarization.

Voter turnout (participant retention)

The experiment was designed as a longitudinal survey over four days, such that all 1,440 Prolific participants who completed the first round were repeatedly re-invited via a message on the platform to complete each subsequent round. Participants had 24 hours to complete each round, with a reminder sent to those who have not done so four hours before the deadline. In total, 77.5% of all participants completed both subsequent voting rounds, 10.6% returned for at least one more round, and 11.9% did not return to vote again. When considering both the two voting rounds and the results/survey round, 74.3% of the participants completed all three rounds, 10.4% returned for only two of these rounds, 4.3% returned for just one, and 11.0% did not return again at all.

We tested whether failure to return in any of the two subsequent voting rounds and the results/survey round was biased by status and network (Table S5). It appears that participants assigned to be poor in the segregated and poor-visible networks are less likely to skip a round. The effects, however, are small (Fig.S11) and unstable if we use alternative operationalizations for the dependent variable: a binary outcome for missing any of the three subsequent rounds or completing all and either a count or binary outcome concerning the two subsequent rounds only. This suggests that the group-level results were largely driven by voters' decisions and not by voter turnout.

Coding the open-ended survey responses

The survey at the end of the experiment elicited free-text responses from participants to two questions: "What was the reason for your voting decisions in the game?" and "Overall, how do you feel about the other group members and their voting decisions?" The responses were manually coded into categories by one of the authors, where the coder was blind to any other information besides the question. The categories were defined and refined iteratively to capture the main motivations and sentiments into as few concepts as possible. Tables S6 and S7 contain description of the categories and list representative responses.

References

[1] Tsvetkova, M., Olsson, H. & Galesic, M. Data and software for "Social networks affect redistribution decisions and polarization". *figshare* (2025). URL `https://doi.org/10.6084/m9.figshare.28676018.v3`.

[2] Schulz, J., Mayerhoffer, D. M. & Gebhard, A. A network-based explanation of inequality perceptions. *Social Networks* **70**, 306–324 (2022). URL `https://www.sciencedirect.com/science/article/pii/S0378873322000296`.

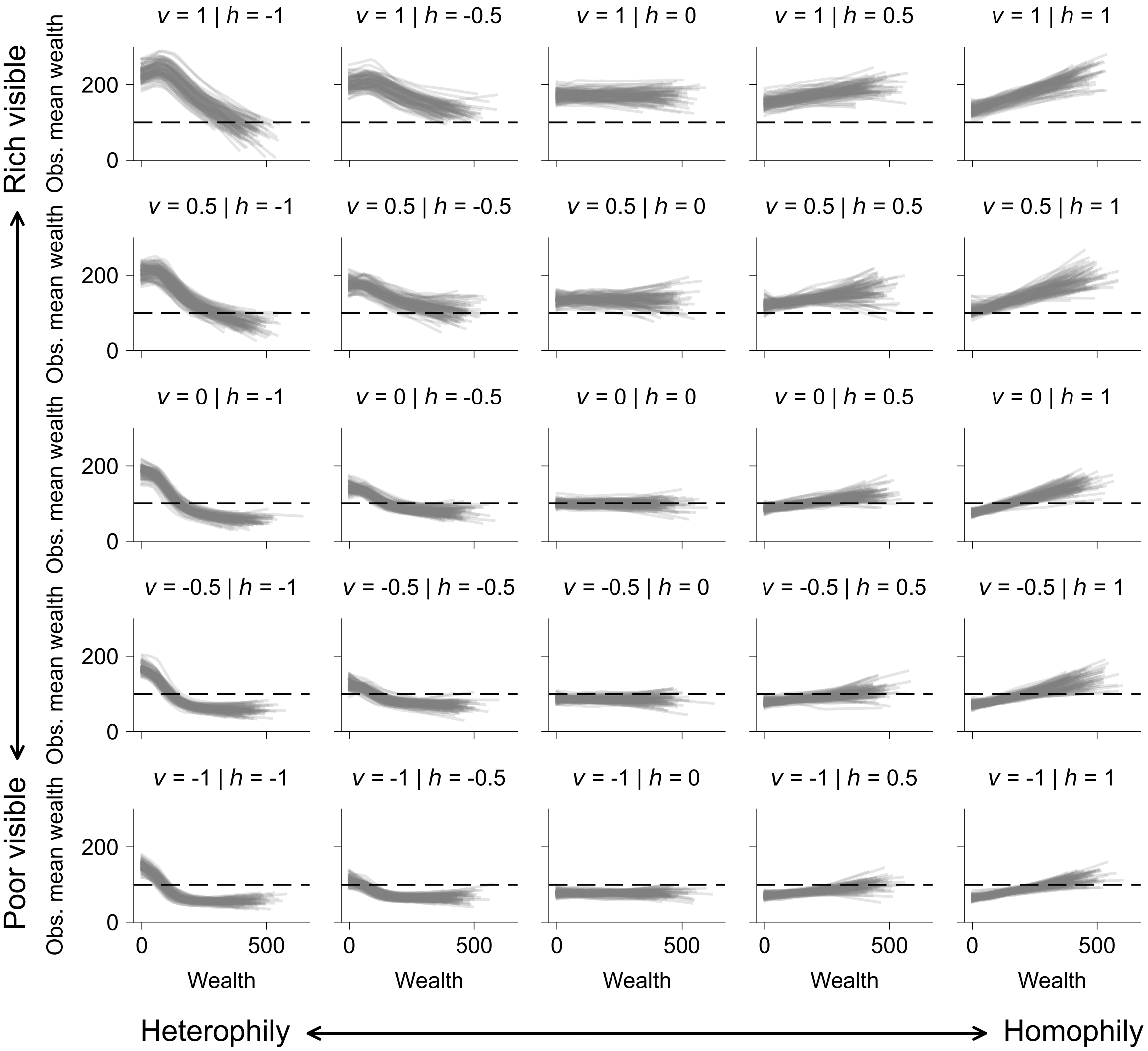

Figure S1: The mean wealth of the neighbors observed by agents of different wealth for varying h (from heterophily on the left to homophily on the right) and v (from higher visibility for the rich on top to higher visibility for the poor on the bottom). The figure shows best-fit Lowess lines for each of 100 simulation runs per parameter combination. The horizontal dashed lines show the mean wealth in the population, which is fixed to 100.

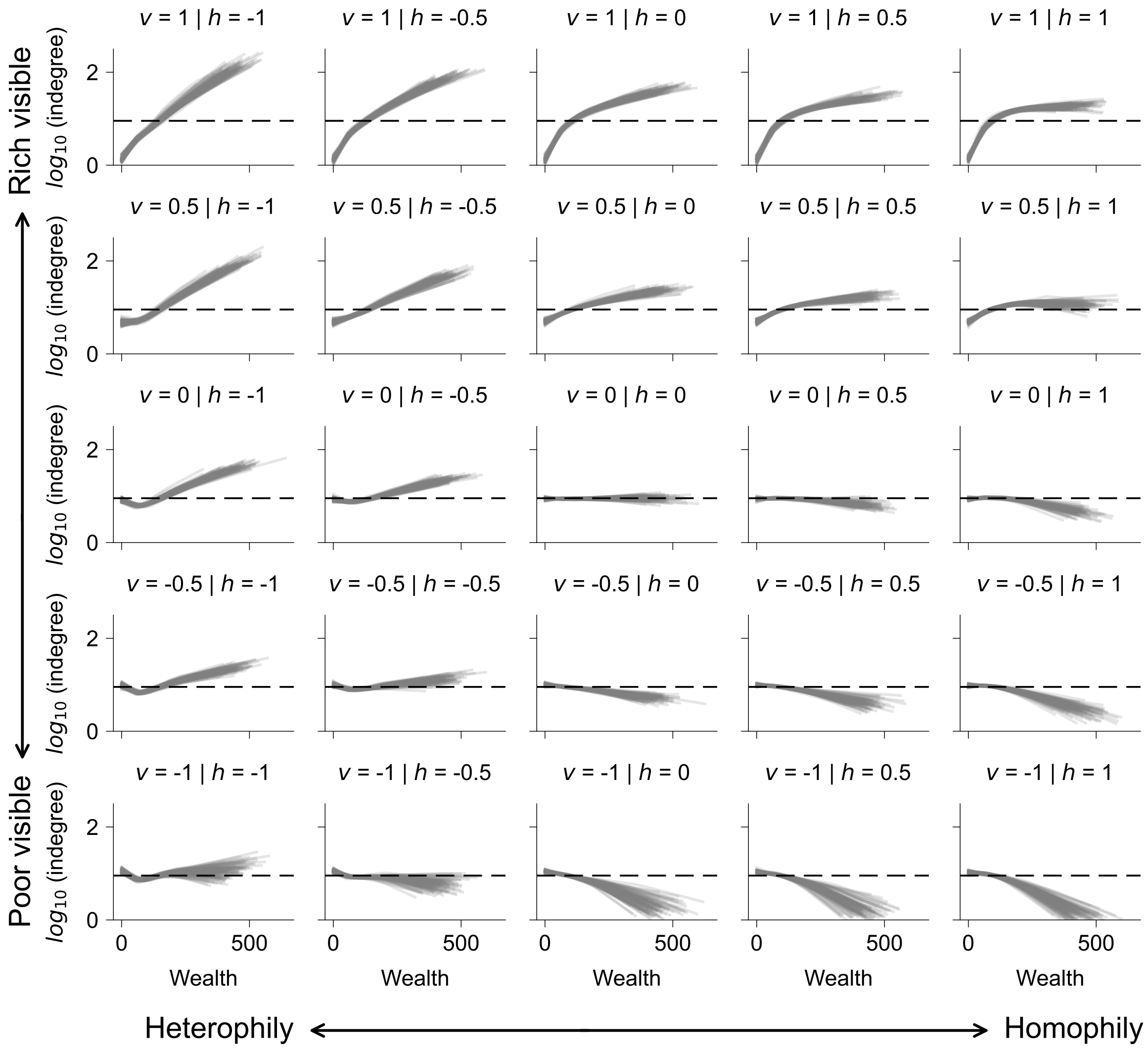

Figure S2: The indegree of agents of different wealth for varying h and v. The figure shows best-fit Lowess lines for each of 100 simulation runs per parameter combination. The horizontal dashed lines show the outdegree (the size of the observation sample), which is fixed in the model to $n = 8$.

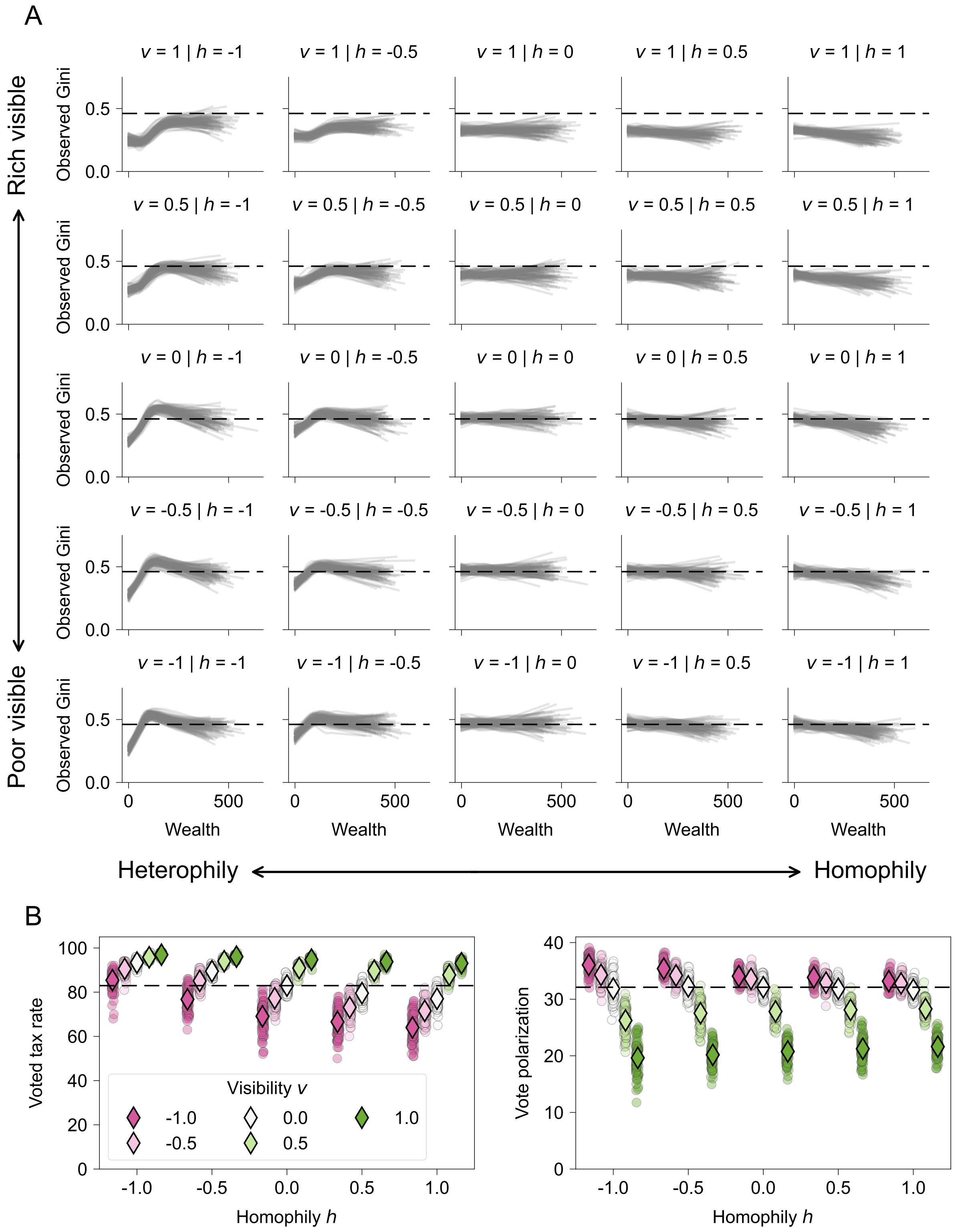

Figure S3: Model results. (A) The observed inequality among neighbors for agents of different wealth for varying assortativity h (from heterophily on the left to homophily on the right) and visibility v (from higher visibility for the rich on top to higher visibility for the poor on the bottom). The figure shows best-fit Lowess lines for each of 100 simulation runs per parameter combination. The horizontal dashed lines show the inequality in the population as a whole, estimated on the complete network where everyone observes everyone else. (B) The voted tax rate (the median vote) and vote polarization (the mean absolute deviation of votes) for varying h and v. Circles show data points from 100 simulation runs, diamonds show means. The horizontal dashed lines show the expected values in the random network with $h = 0$, $v = 0$.

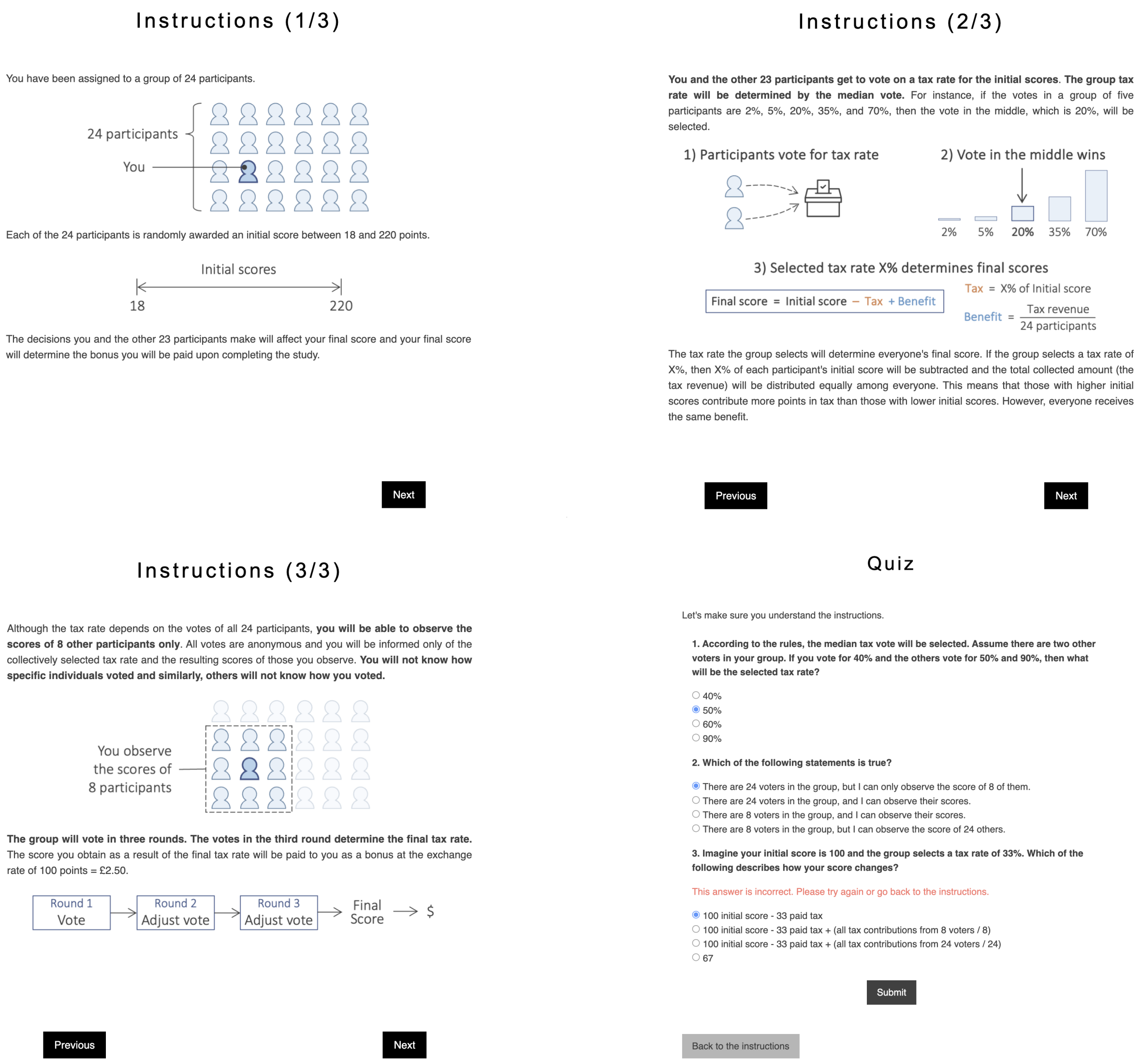

Figure S4: Screenshots of the experiment instructions and quiz.

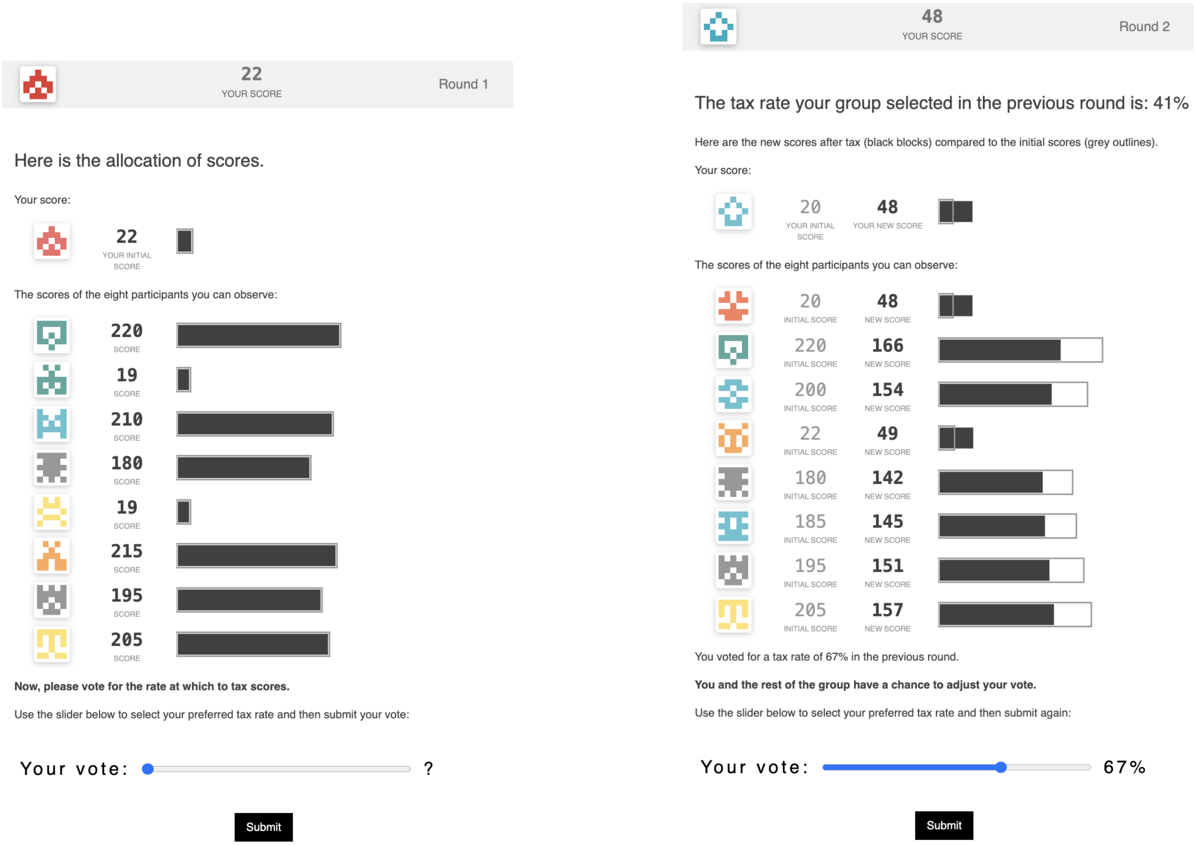

Figure S5: Screenshots of the decision screen in round 1 and round 2 for different players in the same game.

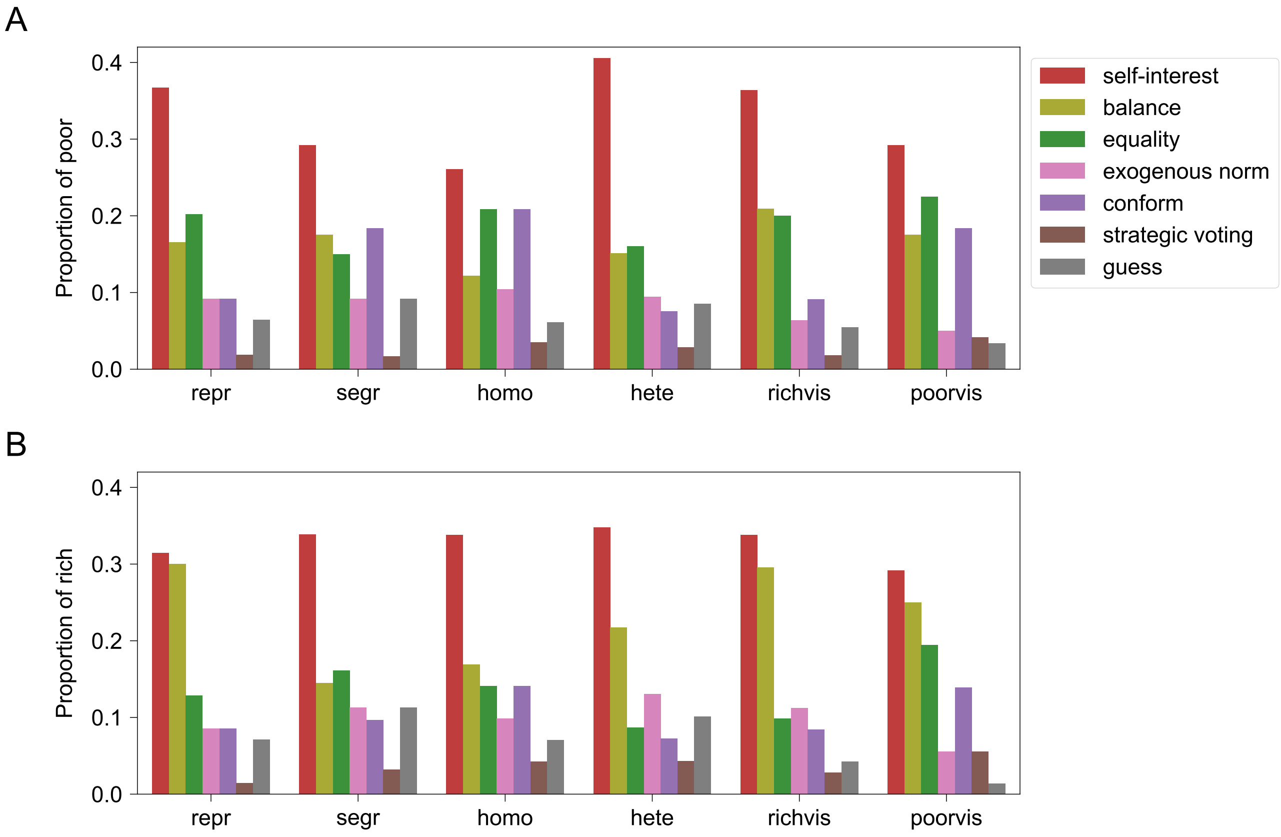

Figure S6: Frequencies of the manually coded categories for the free-text answers to the exit survey question *What was the reason for your voting decisions in the game?* for participants assigned to be (A) poor and (B) rich.

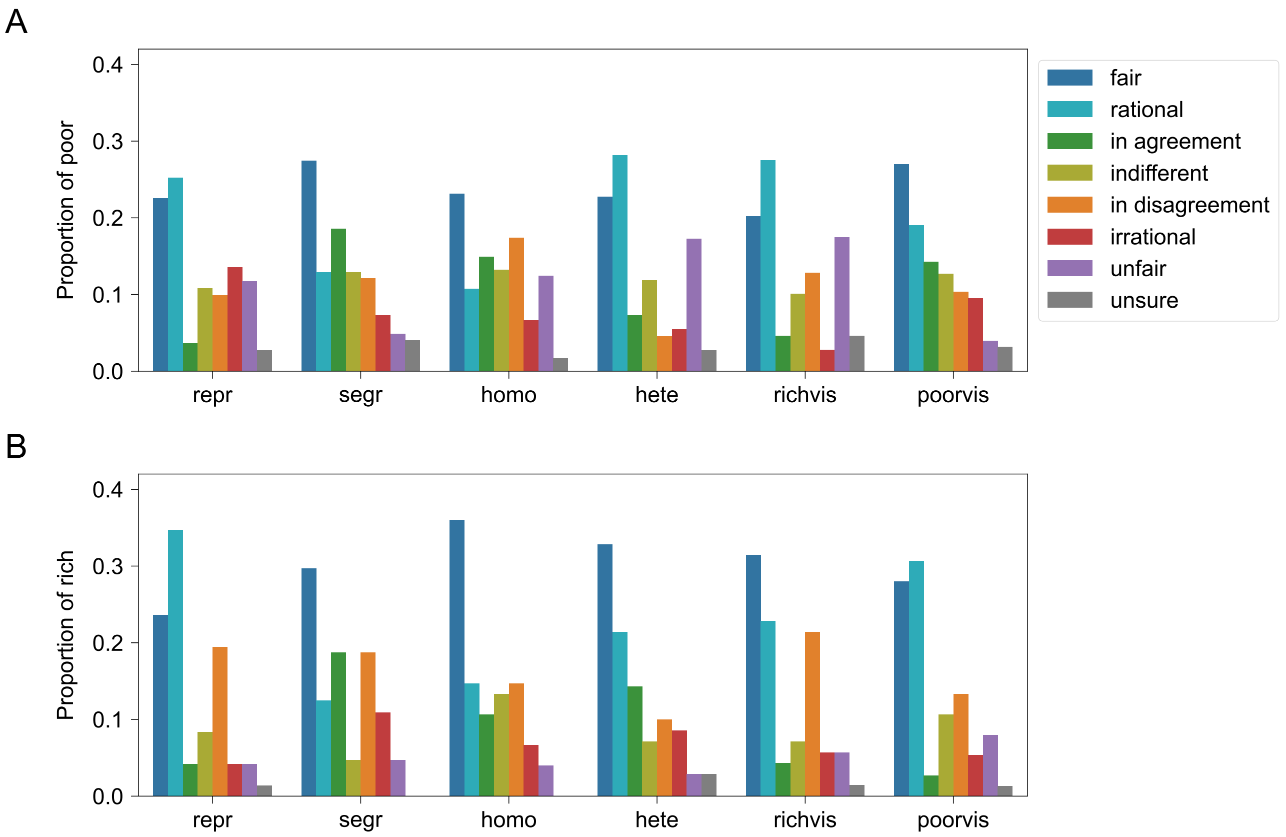

Figure S7: Frequencies of the manually coded categories for the free-text answers to the exit survey question *Overall, how do you feel about the other group members and their voting decisions?* for participants assigned to be (A) poor and (B) rich.

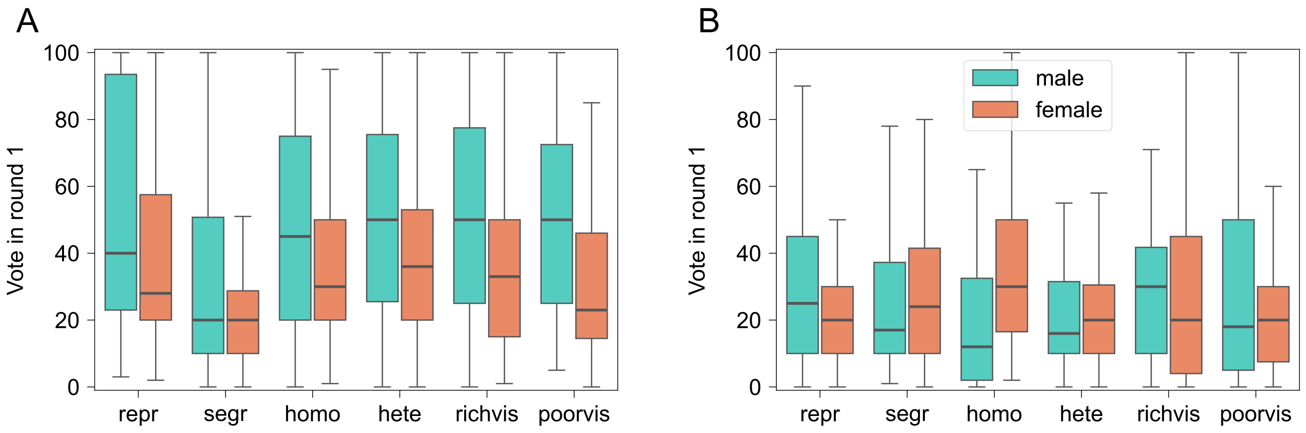

Figure S8: Differences in voting by gender in round 1 for participants assigned to be (A) poor and (B) rich.

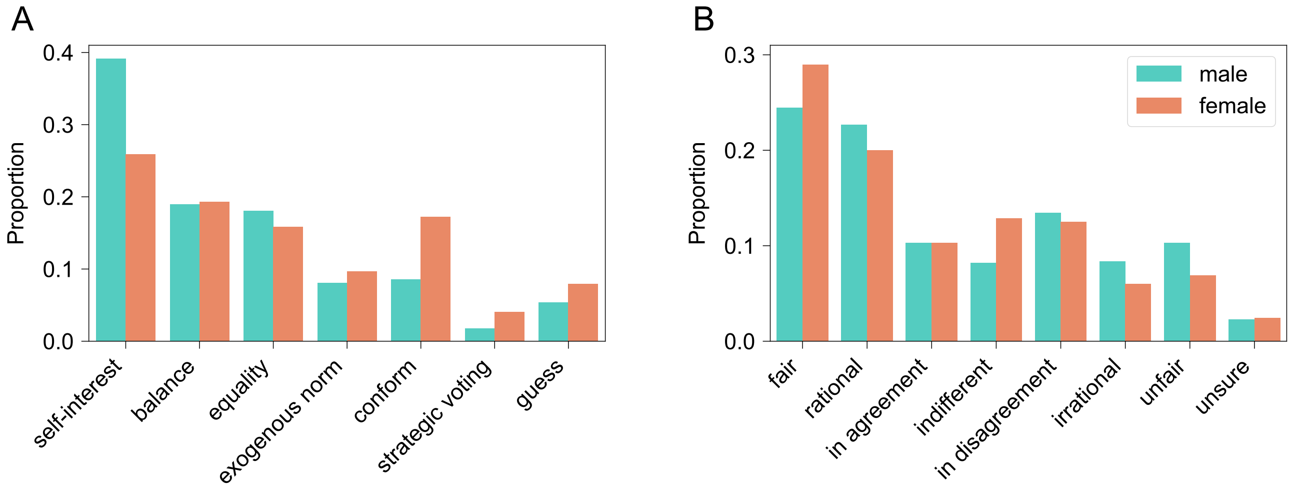

Figure S9: Differences in the answers to the exit survey questions (A) *What was the reason for your voting decisions in the game?* and (B) *Overall, how do you feel about the other group members and their voting decisions?* between men and women.

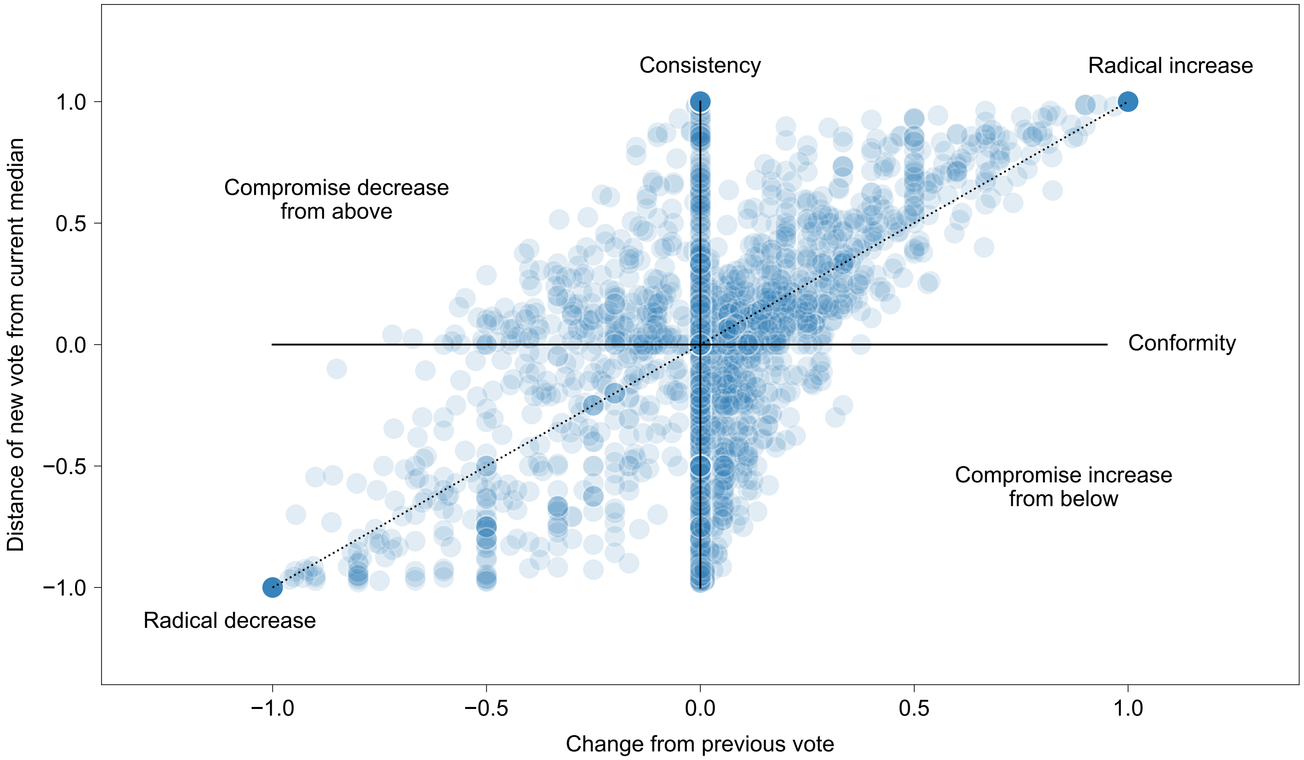

Figure S10: Individual change in vote in rounds 2 and 3 depicted as deviation from the current median vote as a fraction of the possible deviation plotted against the change from the individual's vote in the previous round, again as a fraction of the possible change.

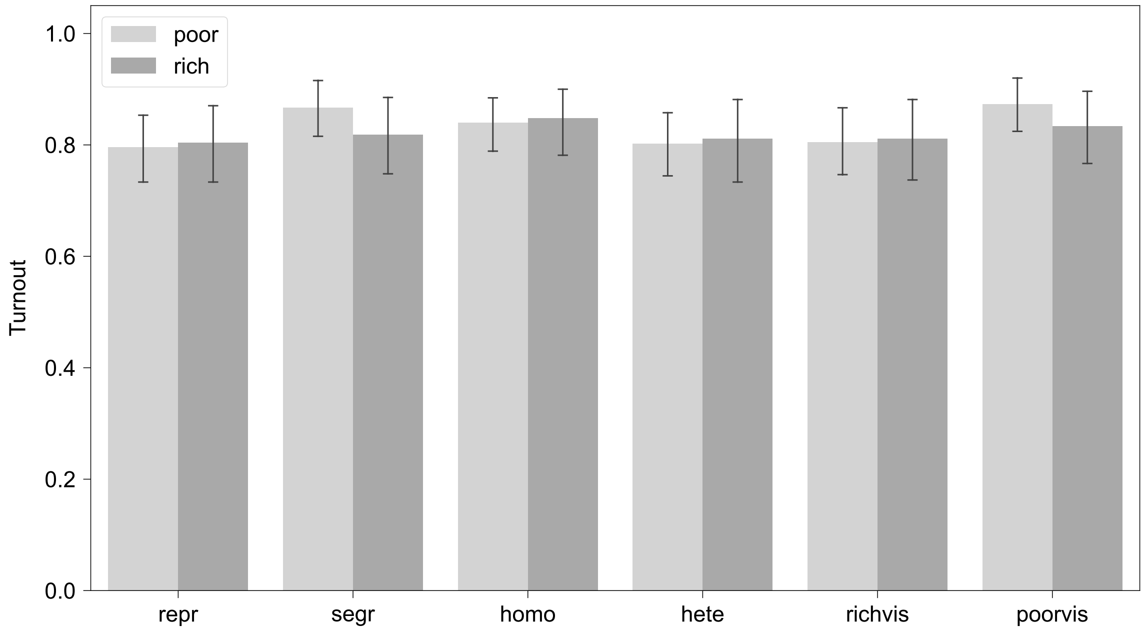

Figure S11: The mean proportion of rounds that participants return for, estimated for round 2, round 3, and the results/survey round.

Table S1: Mann-Whitney U test p-values for the voted tax rate as determined by the median vote. The three results per cell correspond to Rounds 1 to 3 from top to bottom.

Median vote

	segr	homo	hete	richvis	poorvis
repr	0.002**	0.495	0.791	0.063+	0.704
	0.037*	0.304	0.271	0.149	0.567
	0.030*	0.239	0.211	0.119	0.305
segr		0.006**	0.016*	0.001***	0.007**
		0.008**	0.028*	0.004**	0.010**
		0.019*	0.012*	0.004**	0.011*
homo			0.940	0.403	0.362
			1.000	0.879	0.761
			1.000	0.595	0.940
hete				0.288	0.596
				0.762	0.879
				0.705	0.677
rich					0.053+
					0.494
					0.649

*** $p < 0.001$, ** $p < 0.01$, * $p < 0.05$, + $p < 0.1$

Table S2: Mann-Whitney U test p-values for the polarization of votes as measured by the mean absolute standard deviation, the variance, and the kurtosis of the vote distribution. The three results per cell correspond to Rounds 1 to 3 from top to bottom.

Mean absolute deviation of votes

	segr	homo	hete	richvis	poorvis
repr	0.076+	0.678	0.734	0.521	0.623
	0.473	0.307	0.427	0.678	0.427
	0.212	0.385	0.427	0.473	0.212
segr		0.121	0.009**	0.026*	0.121
		0.880	0.064+	0.241	1.000
		0.910	0.021*	0.045*	0.791
homo			0.940	0.521	0.734
			0.076+	0.162	0.910
			0.026*	0.031*	0.850
hete				0.734	0.650
				0.791	0.064+
				0.970	0.045*
rich					0.273
					0.162
					0.064+

Variance of votes

	segr	homo	hete	richvis	poorvis
repr	0.038*	0.473	0.521	0.791	0.273
	0.345	0.273	0.473	1.000	0.273
	0.076+	0.473	0.241	0.385	0.910
segr		0.186	0.064+	0.064+	0.241
		0.970	0.104	0.345	0.850
		0.121	0.005**	0.004**	0.212
homo			0.910	0.734	0.623
			0.121	0.623	0.623
			0.038*	0.054+	0.791
hete				0.678	0.521
				0.521	0.031*
				0.970	0.212
rich					0.473
					0.140
					0.273

Kurtosis of the vote distribution

	segr	homo	hete	richvis	poorvis
repr	0.064+	1.000	0.678	0.064+	0.678
	0.212	0.734	0.273	0.121	0.910
	0.030*	0.239	0.211	0.119	0.305
segr		0.121	0.054+	0.005**	0.031*
		0.273	0.021*	0.007**	0.212
		0.019*	0.012*	0.004**	0.011*
homo			0.345	0.140	1.000
			0.121	0.026*	0.678
			1.000	0.595	0.940
hete				0.212	0.910
				0.970	0.307
				0.705	0.677
rich					0.076+
					0.140
					0.649

*** $p < 0.001$, ** $p < 0.01$, * $p < 0.05$, + $p < 0.1$

Table S3: Coefficients and standard errors (in brackets) from mixed-effects regression model predicting change in individual votes over the three rounds with experiment treatments.

	**Model**
Segregated	−0.637
	(4.151)
Homophilous	−0.061
	(4.147))
Heterophilous	−1.036
	(4.154)
Rich visible	3.423
	(4.155)
Poor visible	1.348
	(4.151)
Assigned poor	13.507***
	(3.631)
Round	0.269
	(0.397)
Segregated:Assigned poor	−14.426**
	(4.959)
Homophilous:Assigned poor	−1.450
	(4.957)
Heterophilous:Assigned poor	3.582
	(4.964)
Rich visible:Assigned poor	−2.849
	(4.965)
Poor visible:Assigned poor	−5.585
	(4.959)
Assigned poor:Round	4.489***
	(0.502)
Constant	25.975***
	(3.105)
Observations	3,823
Participants	1,440
Groups	60
Batches	3
Participant-level variance	630.470
Group-level variance	9.259
Batch-level variance	1.396

*** p ¡ 0.001, ** p ¡ 0.01, * p ¡ 0.05

Table S4: Coefficients and standard errors (in brackets) from simple linear regression model predicting individual votes in the first round with experiment treatments and participant demographics.

	**Model 1**	**Model 2**
Segregated	2.407	2.100
	(4.576)	(4.537)
Homophilous	−1.074	0.788
	(4.489)	(4.453)
Heterophilous	−0.344	0.145
	(4.504)	(4.453)
Rich visible	2.723	4.832
	(4.459)	(4.426)
Poor visible	1.389	1.455
	(4.465)	(4.413)
Assigned poor	22.972^{***}	30.641^{***}
	(4.087)	(4.372)
Age	−0.050	−0.053
	(0.072)	(0.071)
Female	-7.980^{***}	1.928
	(1.658)	(2.640)
Other gender	−2.994	-20.138^{*}
	(7.129)	(12.071)
Politically conservative	-1.761^{**}	-1.731^{**}
	(0.706)	(0.698)
Estimated income percentile	0.084^{**}	0.083^{**}
	(0.036)	(0.035)
Preferred tax rate	0.397^{***}	0.397^{***}
	(0.075)	(0.074)
Segregated:Assigned poor	-19.697^{***}	-19.196^{***}
	(5.757)	(5.697)
Homophilous:Assigned poor	−8.018	−9.218
	(5.727)	(5.665)
Heterophilous:Assigned poor	1.210	0.685
	(5.768)	(5.703)
Rich visible:Assigned poor	−2.652	−4.976
	(5.746)	(5.699)
Poor visible:Assigned poor	−7.720	−7.621
	(5.675)	(5.609)
Female:Assigned poor		-15.826^{***}
		(3.309)
Other gender:Assigned poor		25.174^{*}
		(14.701)
Constant	18.686^{***}	13.721^{***}
	(4.688)	(4.752)
Observations	1,119	1,119
R^2	0.172	0.193
Adjusted R^2	0.160	0.179
Residual Std. Error	26.667 (df = 1101)	26.359 (df = 1099)
F Statistic	13.492^{***} (df = 17; 1101)	13.822^{***} (df = 19; 1099)

*** $p < 0.001$, ** $p < 0.01$, * $p < 0.05$

Table S5: Coefficients and standard errors (in brackets) from mixed-effects Poisson regression model predicting number of absences in the three rounds after the first: round 2, round 3, and the result/survey round.

	**Model**
Segregated	-0.446^{*}
	(0.214)
Homophilous	-0.239
	(0.207))
Heterophilous	-0.026
	(0.201)
Rich visible	-0.044
	(0.201)
Poor visible	-0.481^{*}
	(0.216)
Assigned rich	-0.040
	(0.172)
Segregated:Assigned rich	0.349
	(0.257)
Homophilous:Assigned rich	-0.015
	(0.259)
Heterophilous:Assigned rich	-0.003
	(0.245)
Rich visible:Assigned rich	0.006
	(0.245)
Poor visible:Assigned rich	0.314
	(0.262)
Constant	-0.532^{***}
	(0.142)
Participants	1,440
Groups	60
Batches	3
Group-level variance	0.009
Batch-level variance	0.000

*** p ¡ 0.001, ** p ¡ 0.01, * p ¡ 0.05

Table S6: Description and representative examples of the categories for the free-text responses to the survey question "What was the reason for your voting decisions in the game?".

**Category**	**Description**	**Representative examples**
Self-interest	Aims to maximize own profit	I tried to maximize my chances of scoring highly. It was easier to do this in the later rounds.<br>I am greedy and wanted to maximize the amount of money I kept.<br>My reason for the voting is to earn more bonus<br>I wanted to pay little in taxes so that I could keep my points.
Balance	Concern with fairness, recognizes a tradeoff between own and others' interests	To try and maximize my gains but also to equalize incomes across all<br>I wanted to pick the most fair amount<br>I did not want to be super greedy<br>I wanted to keep as much money I can while still contributing enough taxes that will make a difference.
Equality	Aims equal distribution	I was trying to tax heavily so the taxes with the most had their excess more evenly distributed<br>Since the initial scores were random, It seemed reasonable to make the final payouts close to an even distribution. I chose 70% in order to mostly distribute the scores, but still allow anyone that got a high score to retain a little extra.<br>Spread the wealth<br>I hoped many people would vote for high tax amounts so that the final numbers would be large and then evenly distributed.
Exogenous norm	Refers to taxes they pay, makes a normative statement about taxes in general	chose something close to my state's sales tax.<br>I chose a number close to my real life tax rate.<br>I felt that 25% was fair<br>I've had a history of both having to pay taxes when things are good, and getting taxes back when things are bad (fluctuating income). I don't think people should be punished too much for making money, but I do think there needs to be a reallocating of sorts to people who need it.
Conform	Votes to approximate median vote or expectation of others' votes	I chose an arbitrarily low rate initially and then moved towards the group median in subsequent rounds to reflect the consensus.<br>I was mixing what I thought was fair with what others were voting on.<br>i wanted to keep the tax rates low but it seemed like the others kept pushing to go higher, so i tried to meet in the middle or lower end<br>Mostly based my votes around the majority of what the percentage came to be by the other voters.
Strategic voting	Votes to counteract median vote or expectation of others' votes, votes consistently	I wanted to be on the top half of the rate decisions to try to push the median upwards<br>I was trying to stay low to bring the median down.<br>I tried to vote low in case others decided to put a high rate<br>Maximize median tax rate (I did not expect mine to be implemented)
Guess	Acknowledges confusion, experimentation, or random strategy	I'm not really sure.<br>I tried various choices to see what would happen<br>A bit of experimentation<br>It was somewhat arbitrary. It seemed like a good number at the time

Table S7: Description and representative examples of the categories for the free-text responses to the survey question "Overall, how do you feel about the other group members and their voting decisions?".

**Category**	**Description**	**Representative examples**
Fair	Satisfied with outcome	I feel positive! I think this was a fun experiment.<br>I was fine with it. They didn't seem increase the taxes to much.<br>Their decisions were fine. The final tax rate was better than I expected it to be.<br>I think the group chose well
Rational	Recognizes others act in their own best interest, other's actions are as expected	I guess they did what they thought was best.<br>I'm fine with it, they have their won reasons.<br>I don't have any feelings, I know that peoples intentions to end with a good income was a driving force for decision making<br>The other decisions came in as I expected.
In agreement	Emphasizes similarity and agreement with or among others	I feel like I was probably in a group of mostly like-minded individuals, and seeing the tax rates align with my own choices made me feel like I was around decent people.<br>I think they were thinking very similarly.<br>I feel they were trying to get as much out of it as I was so I am neutral toward them.<br>Don't really have an opinion one way or another. I like that we generally agreed.
Indifferent	Does not express opinion or judgement	No opinion<br>Neutral.<br>I have no feelings about the other group members based on their voting decisions<br>I feel kind of nuetral about it.
In disagreement	Emphasizes disagreement or conflicting interests with others, expresses surprise at others' decisions	It seems many chose a very low tax rate as I was always above the median on choosing what the tax is.<br>I was disappointed that the amounts changed very little because a number of players didn't propose high percentage points.<br>The rates they voted for seemed too low<br>I feel like they could have somehow pushed for more.
Irrational	Accuses others of misunderstanding the game, behaving randomly, acting against their own interests, or being bots	I feel like the other group members did not understand that a higher tax rate would help them rather than hurt them.<br>I suspect they were not real people. But if they were, they were idiots. Any vote other than 0 made no sense.<br>I am perplexed why more people voted around 20<br>All in all, I didn't feel there was that much of an impact in my voting and the voted tax rate seemed like madness.
Unfair	Dissatisfied with outcome, emphasizes unfairness or inequality	I feel they could have voted much higher, and we all could have split a decent profit (aside from the 2 people who had astronomical initial scores).<br>I am a little sad about the outcome.<br>They all seemed to do much better than me.<br>I feel like the points distribution was unfair.
Unsure	Acknowledges confusion or lack of information	Can't really say<br>I don't know<br>No idea<br>They made no sense to me because we couldn't chat.
